# Risk and Opportunity of Using Plastics from Waste Collected in a Yellow Bag

**DOI:** 10.3390/polym12081815

**Published:** 2020-08-13

**Authors:** Jacek Połomka, Andrzej Jędrczak, Sylwia Myszograj

**Affiliations:** 1 Regional Municipal Waste Treatment Plant in Marszów, 68200 Marszów, Poland; J.Polomka@marszow.pl; 2 University of Zielona Góra, Institute of Environmental Engineering, Szafrana 15, 65246 Zielona Góra, Poland; A.Jedrczak@iis.uz.zgora.pl

**Keywords:** plastic waste, yellow bag, reuse, recycling

## Abstract

The article presents the results of research aimed at analysing the share of fractions suitable for recycling and pollutants in waste collected in a yellow bag. The research was carried out in an area inhabited by 200,000 people. The amount of waste collected in the communes in yellow containers in 2016–2019 increased systematically in communes: rural by 75.8, urban–rural by 44.9 and urban by 17.8%. The collection efficiency expressed in the degree of collection was the highest and grew fastest in rural areas (from 25.1 to 35.5%). In cities, it practically did not change (14.4–15.8%). The weight of recyclable components represented on average 39.9% of the weight of yellow bag waste. Plastic bottles (PET) packaging prevailed, the share of which changed from 19.6 to 14.8%, including the share of colourless PET decreasing from 7.9 to 5.8%. At present, revenue from the sale of secondary raw materials obtained from waste collected separately to yellow bags covers only 26% of the costs related to the recycling and recovery of waste delivered to the installation. Support for separate collection of plastics through recovery organisations, i.e., entrepreneurs who are obliged to recover and recycle waste, is symbolic.

## 1. Introduction

Plastics are an important material in many sectors of the economy, but they pose a challenge to sustainable development in terms of waste treatment, resource efficiency and environmental protection. Low recycling rates and high levels of environmental disposal are key issues for environmental technology designers and packaging manufacturers to address.

The production of plastics including thermoplastics, polyurethanes, thermosetting plastics, elastomers, adhesives, coatings and sealing materials (without PET, PP, PA and polyacrylic fibres) in 2018 worldwide amounted to 359 million tonnes and in Europe (EU 28+Norway+Switzerland) to 61.8 million tonnes. The largest manufacturer of plastics (thermoplastics and polyurethanes) in 2018 was China (30.0%). Europe’s share of global plastics production was 17%. European processors’ demand for plastics in 2018 was 51.2 million tonnes. More than 70% of this volume was used by six countries: Germany, 24.6%; Italy, 13.9%; France, 9.4%; Spain, 7.6%; UK, 7.3%; and Poland, 6.8%. The main application sectors for plastics were packaging, 39.9%; construction, 19.8%; automotive, 9.9%; electrical and electronic equipment, 6.2%; household appliances, 4.1%; sport, 4.1%; and agriculture 3.4%. The biggest demand from processors in Europe is for polypropylene (PP), almost 10 million tonnes; PELD, 8.9 million tonnes; PEHD, 6.3 million tonnes; PVC, 5.2 million tonnes; PET, 3.8 million tonnes; and PS 1.9 million tonnes [1,2].

In 2018 (EU 28+Norway and Switzerland), 27.1 million tonnes of plastic waste were separately collected, of which 32.5% was recycled (63% inside the EU, 37% outside the EU), 42.6% was subject to energy recovery and 24.9% was deposited in landfills. A total of 17.8 million tonnes of separately collected plastics were packaging waste, of which 42.0% was recycled, 39.5% was subject to energy recovery and 18.5% was deposited in landfills [1,2]. 

In recent years, half of the plastics collected in the EU for recycling have been exported to countries outside the EU. The reasons for these exports include a lack of capacity, technology or financial resources to treat waste onsite. In the EU, demand for recycled plastics accounts for only 6% of plastic demand and is often limited to low-value or niche applications. China’s import ban on plastic waste in early 2018 surprised the EU and prompted it to speed up regulatory initiatives. 

The administrative regulations in this area are foreseen in the announced EU strategy for plastics (COM/2018/028) [3]. The introduced EU environmental regulations are to contribute to reducing the amount of waste generated and increasing the use of recycled materials (Directive EU 2019/904) [4]. These assumptions are implemented, among others, through the Closed-Circuit Economy Strategy (circularity) and the requirements for the so-called extended producer responsibility, according to which all the costs of packaging waste management are borne by companies introducing packaging to the market. Additionally, an important element of regulatory changes introduced by the EU directive (Directive EU 2019/904) [4] concerning single-use plastic products (Single-Use Plastics Directive), the so-called SUP, will be sanctioned in 2021 to ban and limit the use of 10 disposable plastic products that have environmentally safe alternatives (plates and cutlery, beauty sticks, balloon sticks, polystyrene boxes and cups (EPS), products made of oxo-degradable materials). Another requirement introduced by EU regulations (Directive EU 2019/904) [4] will be an increase in the share of recyclates in PET bottles. Ultimately, by 2030, 30% of PET bottles by weight shall originate from reprocessing. Moreover, by 2025 77% of plastic bottles will have to be separately collected, and by 2030 this percentage shall increase to 90%.

Used packaging can only be recycled if an efficient collection system, segregation and quality assurance of the recyclate are ensured. Administrative interference seems necessary in this area, rationalising the design of packaging from the point of view of a circular economy, as well as limiting its nonusual, purely marketing role [5,6,7], whereas a circular economy could be a possible future solution to the global resource security dilemma [8].

Table 1 gives examples of the use of plastics and the possibilities of their recycling or reuse. It should be noted that thermoplastics are used in typical plastic applications such as packaging (Table 1) but also in other applications such as textile fibres and coatings [9]. Reducing, reusing and recycling represents the fundamental message for resource conservation. Recycling means turning an item into raw materials which can be used again, sometimes for a completely new and other product. Reusing refers to using an item as it is without treatment. This reduces pollution and waste, thus making it a more sustainable process.

The separate waste collection system has been developed very dynamically in many EU countries since the 1990s [11,12], whereas in Poland the process was slow and inefficient. In 2012, about 10% of generated waste was separately collected, and the rate of separate collection of plastics, metals and multimaterial waste per capita did not exceed 5 kg [13]. Numerous studies indicate a high share of pollutants in the waste fractions collected separately, as a result of mistakes made in waste segregation, society’s habituation to the system without separate collection and insufficient environmental education [11,12,14,15,16]. These pollutants hamper treatment processes and reduce the value of the achieved levels of recycling and reuse of waste [17].

In Poland, the method of separate collection of waste is regulated by the Regulation on the detailed method of separate collection of selected waste fractions of 29 December 2016 (Journal of Laws 2016, item 1920) [18]. In accordance with the adopted rules, the yellow bag (container) should be filled with waste which includes metal waste, including metal packaging waste, plastic waste, including plastic packaging waste, and multimaterial packaging waste. In practice, apart from packaging, these containers are filled with nonrecyclable plastics, paper, glass and other components [17].

The article presents the results of research aimed at evaluating the development of separate collection of waste in a yellow bag (container) in 2016–2019, by determining: (i) the changes in the unit waste collection rate; (ii) the effectiveness of separate collection, expressed in terms of the degree of waste collection; (iii) the share of pollutants and recyclable fractions in this waste. 

A financial analysis of the system based on sales of secondary raw materials obtained from yellow bags and costs related to sorting and management of other waste was also performed. 

The research was carried out at the Mechanical–Biological Waste Treatment Plant (MBT) in Marszów (Poland, Lubuskie Voivodeship) that operates a selective waste collection area with a population of 200,000 inhabitants. 

## 2. Installation Characteristics

The MBT installation in Marszów (commissioned in February 2015) The installation serves the residents of 22 municipalities, including 15 municipalities from the Lusatian Union of Municipalities, which owns the installation. According to the population register, 22 municipalities had a total of 201,247 inhabitants in 2019, of which 56,347 were in 12 rural municipalities, 59,621 in five urban–rural municipalities and 85,279 in five urban municipalities. The smallest rural commune had 2255 inhabitants, and the largest of the towns was inhabited by 37,465 people. 

The “yellow bag” waste treatment plant is equipped with a bag breaker, a three-fraction sieve with an active length of 11 m, two ferromagnetic separators, one eddy-current separator for aluminium, four photo-optical separators, a ballistic separator and five sorting cabins. For the production of RDF, there is a slow-running twin-shaft mill and a fast-running single-shaft mill. 

The waste is then transferred by means of a wheel loader to the bag breaker and then to a drum screen, where the 0–80 mm fraction, used to produce substitute fuels, is mechanically separated. The remaining 80–260 mm stream is directed to a ballistic separator, which separates the waste into a 2D flat fraction and a 3D spatial fraction. The ballistic separator has 40 mm diameter holes, which are used to screen out the contamination for RDF production. The recyclable waste, as well as waste potentially containing chlorine, which cannot be used for fuel production, is separated mechanically and manually from the remaining waste. Other waste containing, among others, multilayer plastic packaging, toys, crates, pots, elements of WEEE equipment and dirty packaging, goes as “ballast” to the RDF production line. Lines configured in this way enable separation of the following material fractions: colourless PET, blue PET, green PET, PET mix, household chemistry packaging, PEHD film, PELD film, steel cans+steel scrap, aluminium cans+aluminium scrap, multimaterial packaging (of liquid food), paper mix, PP+PE (yoghurt and fat packaging).

Mass and material balances of the processing of waste collected in yellow bags (containers) were carried out between 2016 and 2019, in March. Waste from the area of bags collection, after being delivered to the installation, was mixed and its storage was analysed on the industrial line. The weight of the measurement series was 20,600, 51,660, 34,480 and 28,740 kg in subsequent years.

## 3. Research Results and Discussion

### 3.1. Amount of Waste Collected

**Figure 1:**
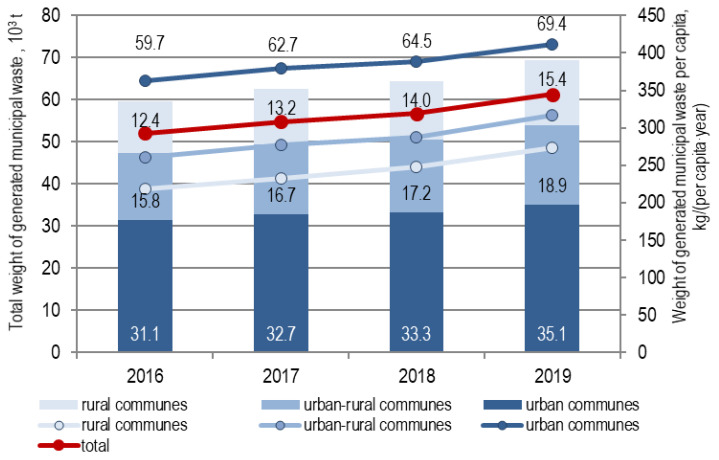
Total weight of generated municipal waste (bars) and per capita (lines) delivered to the Mechanical–Biological Waste Treatment Plant (MBT) installation in Marszów, in 2016–2019.

In 2016–2019, the amount of municipal waste generated in the area served by the MBT installation in Marszów increased from 59,698 to 69,411 t, and per capita from 293 to 345 kg (an increase of 18%) (Figure 1). 

**Figure 2:**
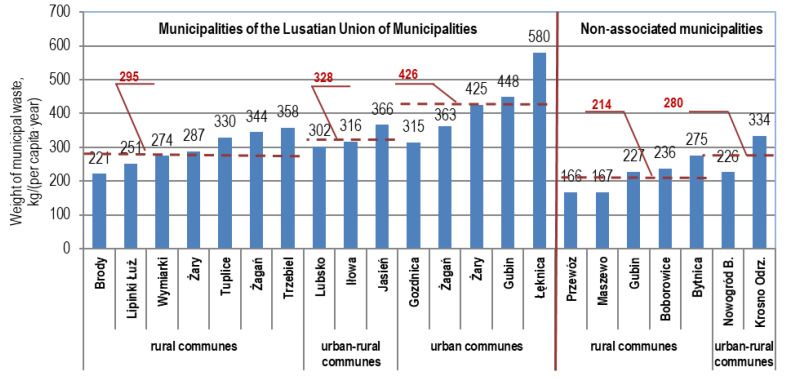
Weight of municipal waste generated per capita according to the population register in 2019 (Municipalities of the Lusatian Union of Municipalities and nonassociated municipalities) by collection area.

In 2019, 56,773 t of municipal waste was delivered to the MBT installation in Marszów from the municipalities belonging to the Lusatian Union of Municipalities, which gives a ratio of 366 kg/per capita/y. This indicator was: in rural communes 295 kg, in urban–rural communes 328 kg and in urban communes 426 kg (Figure 2).

In 2019, 12,638 t of municipal waste was delivered to the plant from nonassociated communes, corresponding on average to 274 kg/per capita/y, including 214 kg in rural communes and 280 kg/per capita/y in urban–rural communes.

**Figure 3:**
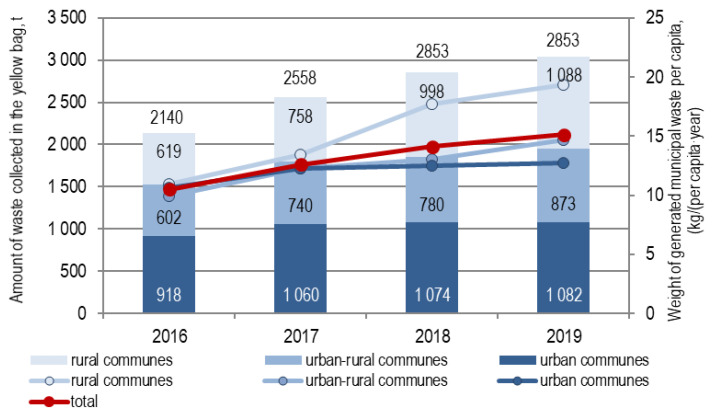
Amount of waste collected in the yellow bag in 2016–2019 by collection area.

The amount of waste collected separately into the yellow container (bag) in the Municipalities of the Lusatian Union of Municipalities steadily increased between 2016 and 2019 (Figure 3). During these four years, the amount of collected waste increased: in rural communes by 75.8%, in urban–rural communes by 44.9%, and in urban communes by 17.8%. 

**Figure 4:**
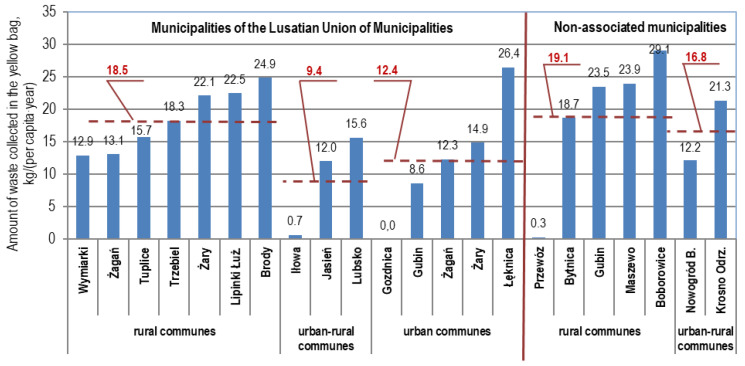
Weight of waste collected separately in the yellow sack (container) per capita according to the 2019 population register (Lusatian Union of Municipalities and nonassociated communes) with a breakdown by collection area.

The collection rate of this waste per capita/y taking into account all waste delivered to the Marszów installation, without division into the collection area, was, respectively: in 2016, 10.5 kg/(per capita/y); in 2017, 12.6; in 2018, 14.1; and 2019, 15.1 kg/(per capita/y). On average in Poland in 2018, according to the Statistics Poland (CSO, 2019) [13], the amount of separately collected waste into the yellow container (bag) per capita was 8.9 kg. The values of the indicator per capita in 2019 with a breakdown into the collection area are shown in Figure 4. This indicator was, on average, 18.5 kg/(per capita/y) in rural, 9.4 kg/(per capita/y) in urban–rural and 12.4 kg/(per capita/y) in urban municipalities associated with the Lusatian Union of Municipalities. For nonassociated municipalities, the indicators were higher: rural municipalities 19.1 kg/(per capita/y) and urban–rural municipalities 16.8 kg/(per capita/y), respectively. 

### 3.2. Efficiency of Separate Collection into the Yellow Bag

The efficiency of separate collection of waste in the yellow bag was assessed by determining the degree of waste collection. This indicator is determined by the quotient of the amount of waste collected in the yellow bag and its overall potential in the generated municipal waste, expressed as a percentage. The share of waste collected to the yellow bag in municipal waste was determined on the basis of studies of its morphological composition. In 2016, 2017, 2018 and 2019 it was: waste from rural areas—19.8 19.8, 19.9 and 20.0%, and in urban waste—20.3, 20.5, 20.5 and 20.6%. For urban–rural communes, the value of the share was calculated as a weighted average of the indicators for cities and villages, assuming the number of inhabitants as weights. 

**Figure 5:**
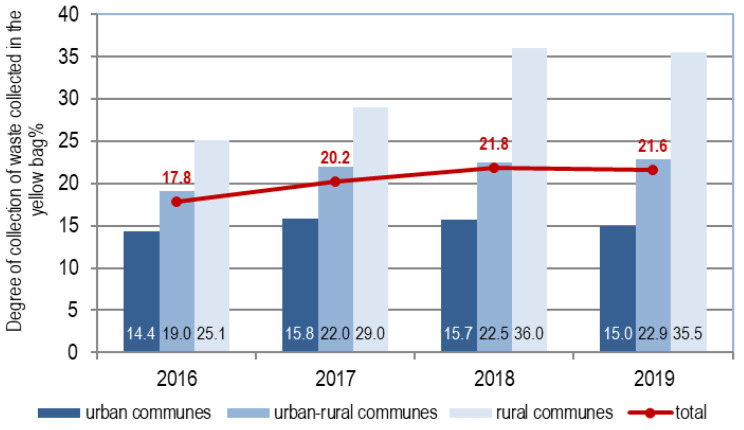
Degree of collection of waste collected in the yellow bag in 2016–2019.

Changes in the degree of waste collection in 2016–2019 are shown in Figure 5. 

In 2016–2018, the collection rate in the region increased from 17.8 to 21.8%, and in 2019 it decreased to 21.6%. The efficiency of waste collection in the yellow bag was the highest and the fastest growing in rural areas. It practically did not change in cities during this time. Great diversity in the effectiveness of separate collection was also found in municipalities both associated and nonassociated with the Association of Municipalities. In communes associated with the Association, the collection rate in 2016–2019 increased by 12% (from 16.8 to 18.8%), and in the remaining communes by 50% (from 22.8 to 34.4%). 

### 3.3. Morphological Composition of Waste

**Figure 6:**
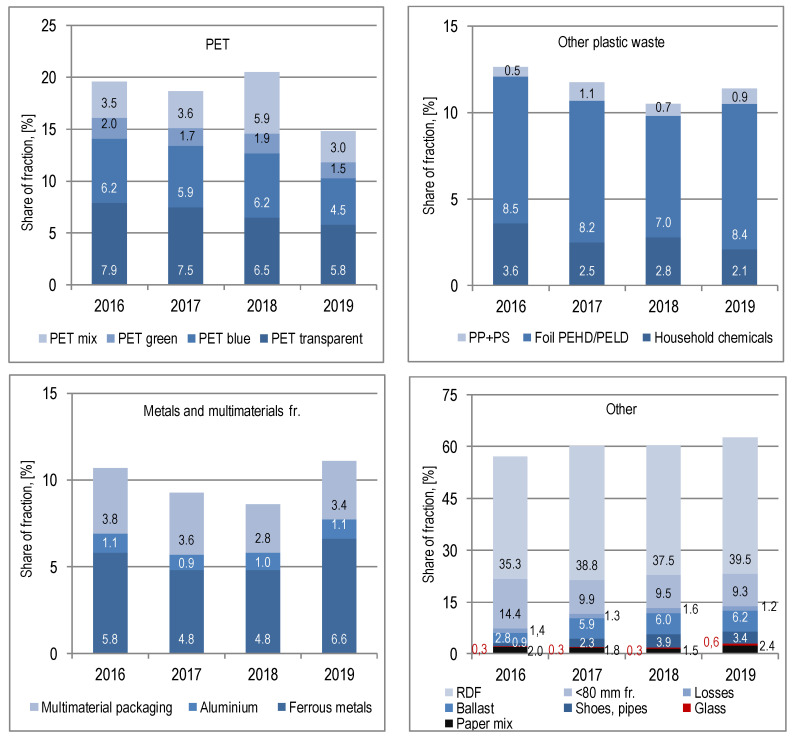
Material composition of waste collected separately in the yellow bag in 2016–2019.

Figure 6 shows the material composition of waste collected in the yellow bag, divided into recoverable and recyclable fractions and other fractions obtained by mechanical sorting, in 2016–2019. 

Plastic, sheet steel, aluminium and multimaterial packaging should be placed in the yellow bag. Unfortunately, people throw everything that resembles plastics and metals into this bag (container), not just packaging made of these materials. This type of waste includes, among others, plastic toys, sewage pipes, water pipes, water pipes, skirting boards, window profiles, PVC carpets, garden equipment (chairs, tables, pots, umbrellas, footwear, WEEE equipment, car parts and others (Figure S1 Supplementary Materials)).

From the waste delivered to the installation in Marszów in the yellow bag, in the first stage of processing in a drum sieve, a 0–80 mm fraction is separated, which goes for fuel production. It accounted for 9.3 to 14.4% (10.8% on average) of the weight of waste to be sorted (Figure 6, Figure S2). Next, contaminants are separated on the ballistic separator (fraction < 40 mm), which also goes for fuel production. They represented 1.3% of the weight on average. The remaining waste is separated mechanically and manually (Figure 6, Figure S2 Supplementary Materials): (i) packaging waste sent for recycling; (ii) waste potentially containing chlorine (shoes, sewage pipes, carpets, skirting boards, window profiles, etc.) that cannot be used for fuel production. The average share in the waste from the yellow sack was 2.6% in 2016–2019; (iii) waste containing, among others, multilayer plastic packaging, toys, crates, pots, elements of WEEE equipment, dirty packaging, the so-called “ballast”, which is directed to a fuel production line, with an average share of 5.2%; (iv) RDF, directed to fuel production, with an average share of 37.8%. 

The weight of recyclable components represented 39.9% of the weight of yellow bag waste on average. The share of colourless PET in yellow bag waste decreased in 2016–2019 from 7.9 to 5.8%, and the shares of blue, green and mix PET remained at a comparable level in 2016–2018, with a clear decrease in 2019. An increase in the number of coloured packaging, coloured in the mass of the polymer with intensive colours, as well as with PVC wrappers, was found.

Unfortunately, the variety of colours of packaging made of polyethylene terephthalate causes a significant drop in prices for the raw material obtained from recyclers. There is practically no interest in PET bottles with labels made of PVC. The presence of PVC in PET packaging causes a drastic decrease in the material properties, making its reuse and final disposal impossible. This is particularly relevant for energy recovery techniques [9,19,20,21].

However, removing PVC packaging from the stream of packaging is costly due to the required accuracy, as little as 0.005% of the admixture of PVC causes a loss of PET properties [22].

The packaging industry uses polyethylene (PE) and PP polypropylene, and PS polystyrene to a lesser extent. PS, like PVC, is a difficult material to recycle. As a result, there are no recycling installations for these plastics or separate collection systems as for PE or PP [23,24]. The share of PEHD and PELD film in the analysed waste collected separately into the yellow bag was between 7.0 and 8.5%. The PEHD film dominated. PP+PS packaging (mainly for yoghurt) constituted 0.8% of collected waste weight on average. The share of household chemistry packaging (packaging made of PEHD, LDPE, PP, PS) did not show a tendency to change during the research period and amounted on average to 2.8%.

Malinowski et al. [16], in the research on changes in morphology (manual sorting) of waste collected separately into the yellow bag during the year from the area of three rural communes, found similar shares of particular commercial fractions in recyclable plastics to the research in Marszów. However, the weight of recyclable plastics was 1.7 times higher, which was due to manual sorting of the whole weight of samples. 

The research conducted by Lewandowska et al. [25] also confirms the determined morphological composition of plastic waste in Poland. The morphological composition of municipal waste collected selectively in two types of communes was analysed: large rural communes and rural–urban communes. In both communes, colourless PET waste and the coloured LDPE film had the largest share in plastic waste. The PEHD film had the smallest share.

A similar share of individual types of plastics in separately collected packaging waste was found in the UK on the basis of annual surveys under the project: An assessment of the technical, environmental and economic viability of recycling domestic mixed plastics packaging waste in the UK. PET packaging had the largest share, followed by PP, PS and PVC. From the plastics in the household bin, the total nonbottle plastics including films, bags and other packaging, comprise 5.2%. The specific nonpackaging fraction is estimated at 1.4% of the bin by weight. Nonpackaging plastics typically include children’s toys, cooking utensils, piping and cabling, household fixtures and fittings, electronic equipment and furniture [26,27].

For the remaining material fractions, such as multimaterial packaging (average share of 3.4%), steel and aluminium scrap (average share of 5.5% and 1.0%, respectively), no significant changes were found in the quantity collected in the yellow bag and delivered to the installation in Marszów in 2016–2019. 

In addition, the tested waste delivered to the installation in Marszów included glass (0.4%) and paper mix (1.9%), which can also be partially recycled but should not be collected in the yellow bag. In the study of the annual analysis of the morphology of waste collected separately into the yellow bag from the area of three rural communes, Malinowski et al. [16] found that the share of glass in the stream of separately collected plastics was, on average, as high as 3.7% and 6% of paper and cardboard. 

### 3.4. Cost-Effectiveness of the System

The system should be as cost-effective as possible. If the same level of recycling could be achieved by other, less costly means, it should be switched to such a measure. Beyond this general principle, decisions on how comprehensive the system should be and what financial obligations should be imposed on the industry are of a political nature and are often factors that limit the advancement of the system. 

Table 1 presents the costs and revenues related to the management of waste collected separately into the yellow bag in the MBT installation in Marszów in 2016–2019.

The amounts of waste collected in the yellow bag increased by 42% between 2016 and 2019 (Table 2). The recovery rate remains very high for all years, over 95%. The demand for subsidies per tonne collected increased by 30.4% and per tonne of recovered packaging by 34.3%. The need for cofinancing per recovered percentage point increased very strongly, as much as by 91.0%.

At present, revenue from the sale of secondary raw materials obtained from waste collected separately in yellow bags covers only 26% of costs related to recycling and recovery of waste delivered to the Marszów installation, and recovery organisation subsidies 0.3%. Support for separate collection of plastics into yellow bags through recovery organisations, i.e., entrepreneurs who are obliged to recover and recycle waste, is symbolic.

If a general deposit system for plastic bottles (PET) and aluminium cans is introduced, the revenue obtained for the sale of other raw materials will cover the processing costs by only 5%.

## 4. Conclusions

The effectiveness of separate collection of waste in the yellow bag, expressed in terms of quantity per capita and the degree of collection, increased steadily between 2016 and 2018. However, there is still a very large variation in collection levels within the region.

Mistakes made in waste segregation, as well as society’s habituation to a system in which waste was not collected by fractions, result in a large share of pollutants in waste collected separately into the yellow bag. Plastic packaging waste is particularly problematic here due to the high volume to weight ratio, it is not an attractive material for collection and recycling, and the costs of transport, reprocessing and the value of the raw material have an impact on the final price of recycled polymer material.

As shown by analyses of various systems in Europe [28], it is recommended to focus on separate collection of plastic fractions based on the quality of the recyclables and not based on waste amounts. Even if this means that less material is collected, the quality of materials collected will be much better (less “sorting mistakes”) and therefore such materials actually are suitable for recycling. A focus on separate collection of certain plastic fractions only (such as plastic bottles) allows for high efficiency of sorting plants (fewer fractions). Over the last several years, some countries (Slovakia, Poland, Czech Republic, Latvia, Lithuania) had significantly lower recycling rates accompanied by low landfill taxes. The evaluation of waste production and recycling can be used for government policy in the area of waste management, as well as for individual communities dealing with communal waste [29].

The recycling system in Poland needs to be changed in terms of improving the efficiency of separate collection and improving its economic efficiency. To do this, it should:

(1) Change the existing mechanisms of Extended Producer Responsibility, which turned out to be ineffective in Poland, the system must receive many more financial resources;

(2) Introduce more effective implementation of eco-design in the production of products (not only for packaging applications), this will have a positive impact not only on recycling levels, but also on the quality of recycled plastics;

(3) Introduce mechanisms that will additionally burden the producers of nonrecyclable packaging;

(4) Make certain products compulsory for a minimum content of recycled materials;

(5) Introduce standardization and certification of the quality of recyclates. This will level the opportunities and open new markets for recycled plastics with uniform and repeatable properties, while ensuring good characteristics, quality and safety of products.

Nowadays, unfortunately, knowledge has to be quickly verified due to the impact of pandemics and epidemics on tracking the life cycles of various plastic products, especially those needed for personal protection and healthcare. The energy and environmental footprint of these product systems have increased quickly in response to the increase in COVID-19 cases worldwide with critical hazardous waste [30].

## Figures and Tables

**Table 1:** Types of plastics and their recycling and reuse potential [10].

Plastic Type	Example of Applications	Assigned Number and Recycling and Reuse
PET	Salad dressing containers, processed meat packages, plastic soft drink and water bottles	Recycled but not reused
PEHD	Milk bottles, shampoo bottles, detergent bottles, oil jerry cans and toys	Reusable and recyclable
PVC	Fruit plastic packing, sweet trays and blister packing	Not recyclable nor reusable (although it is not recommended to reuse PVS, it can be repurposed for other functions excluding food and children’s use)
LDPE	Bread bags, frozen food bags, squeezable bottles, fibre, bottles, clothing, furniture, carpet, shrink-wraps and garment bags	Reusable but rarely recyclable
PP	Margarine and yoghurt containers, caps for containers and wrapping to replace cellophane	Reusable but rarely recyclable
PS	Egg cartons, fast food trays and disposable plastic silverware	Reusable but rarely recyclable
Other	This includes an item which is made with a resin other than the six listed above or a combination of different resins	None. Not recyclable nor reusable except those with polylactic acid (pla) coding underneath

**Table 2:** Balance of costs and revenues for the management of waste collected separately into the yellow bag in the MBT installation in Marszów 2016–2019 (PLN—monetary unit in Poland).

Parameter	Unit	2016	2017	2018	2019
System operating cost	thousand PLN	2448.4	3088.8	3640.6	4108.8
Value of sales of raw materials	thousand PLN	708.4	794.5	1053.7	876.9
Value from recovery organisation subsidies	thousand PLN	7.2	9.6	11.1	19.5
Total income	thousand PLN	715.6	804.2	1064.8	896.5
Need for funding	thousand PLN	1733	2285	2576	3212
Waste collected	tonnes	2140	2558	2853	3043
Material recovery (recycling)	tonnes	962	1061	1172	1208
Energy recovery (fuel)	tonnes	1153	1429	1558	1712
Recovery rate	%	98.8	97.4	95.7	95.9
Need for funding per tonne collected	PLN/t	810	893	903	1056
Change in funding needs, per tonne collected	%	−	10.3	1.1	16.9
Need for funding per tonne recovered	PLN/t	819	917	943	1100
Change in need for funding, per tonne recovered	%	−	11.9	2.8	16.6
Need for funding per the recovered share, %	PLN/%	17,533	23,459	26,907	33,480

## Supplementary Materials

The following are available online at https://www.mdpi.com/2073-4360/12/8/1815/s1, Figure S1: Yellow bag waste; Figure S2: Waste fractions separated on the process line.

## References

[1] Plastics–the Facts 2019. www.plasticseurope.org.

[2] Eurostat. https://ec.europa.eu/eurostat/data/database.

[3] COM/2018/028 final: Communication from the Commission to the European Parliament, the Council, the European Economic and Social Committee and the Committee of the Regions; a European Strategy for Plastics in a Circular Economy. www.eur-lex.europa.eu.

[4] Directive (EU) 2019/904 of the European Parliament and of the Council of 5 June 2019 on the Reduction of the Impact of Certain Plastic Products on the Environment (PE/11/2019/REV/1). http://data.europa.eu/eli/dir/2019/904/oj.

[5] Posłuszny K. (2018). Determinants of circular economy development in plastics. Gospod. Prakt. Teor..

[6] Methodology: EPA’s Facts and Figures on Materials, Waste and Recycling; Plastics: Material-Specific Data. Report Epa. www.epa.gov.

[7] Greco A., Frigione M., Maffezzoli A., Marseglia A., Passaro A. (2014). A Perspective on the Prowaste Concept: Efficient Utilization of Plastic Waste through Product Design and Process Innovation. Materials.

[8] Cucchiellaa F., D’Adamoa I., Gastaldia M., Kohb S.C.L., Rosac P. (2017). A comparison of environmental and energetic performance of European countries: A sustainability index. Renew. Sustain. Energy Rev..

[9] Brems A., Baeyens J., Dewil R. (2012). Recycling and recovery of post-consumer plastic solid waste in a European context. Therm. Sci..

[10] Seaman G. (2012). Plastics by the Numbers, Eartheasy. https://learn.eartheasy.com/articles/plastics-by-the-numbers.

[11] Dahlen L., Vukicevic S., Meijer J.-E., Lagerkvist A. (2007). Comparison of different collection systems for sorted household waste in Sweden. Waste Manag..

[12] Rada E.C., Zatelli C., Cioca L.-I., Torretta V. (2018). Selective collection quality index for municipal solid waste management. Sustainability.

[13] Central Statistical Office, Statistical Information and Elaborations. www.stat.gov.pl.

[14] Larsen A.W., Merrild H., Moller J., Christensen T.H. (2010). Waste collection systems for recyclables: An environmental and economic assessment for the municipality of Aarhus (Denmark). Waste Manag..

[15] Rada E.C., Zatelli C., Mattolin P. (2014). Municipal solid waste selective collection and tourism. WIT Trans. Ecol. Environ..

[16] Malinowski M., Grzelec K., Gutwin M. (2018). Analysis of Impurities in Selectively Collected Plastic Waste-Case Study. Infrastructure and Ecology of Rural Areas, No II/1/2018, POLISH ACADEMY OF SCIENCES, Cracow Branch. www.researchgate.net.

[17] Gallardo A., Bovea M.D., Colomer F.J., Prades M. (2012). Analysis of collection systems for sorted household waste in Spain. Waste Manag..

[18] Regulation of the Minister of the Environment of 29 December 2016 on the Detailed Method of Separate Collection of Selected Waste Fractions (Journal of Laws, item 19; uniform text Journal of Laws 2019 item 2028). http://www.isap.sejm.gov.pl.

[19] Panda K., Achyut I., Singh R.K., Mishra D.K. (2010). Thermolysis of waste plastics to liquid fuel: A suitable method for plastic waste management and manufacture of value-added products—A world perspective. Renew. Sustain. Energy Rev..

[20] Everaert K., Baeyens J. (2001). Correlation of PCDD/F emissions with operating parameters of municipal solid waste incinerators. J. Air Waste Manag. Assoc..

[21] Fazli A., Rodrigue D. (2020). Waste Rubber Recycling: A review on the evolution and properties of thermoplastic elastomers. Materials.

[22] (2016). The New Plastics Economy-Rethinking the Future of Plastics. World Economic Forum, Ellen MacArthur Foundation and McKinsey & Company. http://www.ellenmacarthurfoundation.org/publications.

[23] Drzyzga O., Prieto A. (2018). Plastic waste management, a matter for the ‘community’. Microb. Biotechnol..

[24] Al-Salem S.M., Lettieri P., Baeyens J. (2009). Recycling and recovery routes of plastic solid waste (PSW): A review. Waste Manag..

[25] Lewandowska A., Chołody M., Rawski J., Wiącek S., Wójcik G., Bogucka-Wójcik B. (2016). Skład morfologiczny wybranych frakcji odpadów komunalnych zbieranych w sposób selektywny w okresie letnim. Inżynieria Ekol..

[26] Ragaert K., Delva L., Van Geem K. (2017). Mechanical and chemical recycling of solid plastic waste. Waste Manag..

[27] (2006). WRAP, Material Change for a Better Environment: Domestic Mixed Plastic Packaging Waste Management Options. http://www.wrap.org.uk/.

[28] Seyring N., Dollhofer M., Weißenbacher J., Herzog M., McKinnon D., Bakas I. (2015). Assessment of separate collection schemes in the 28 capitals of the *EU*. BiPro. Novembro.

[29] Taušová M., Mihaliková E., Cˇulková K., Stehlíková B., Tauš P., Kudelas D., Štrba L. (2019). Recycling of communal waste: Current state and future potential for sustainable development in the EU. Sustainability.

[30] Klemes J.J., Fan Y.V., Tan R.R., Jinag P. (2020). Minimising the present and future plastic waste, energy and environmental footprints related to COVID-19. Renew. Sustain. Energy Rev..

