# Supplementary material for: Risk and Opportunity of Using Plastics from Waste Collected in a Yellow Bag
Source: Polymers (Basel). 2020 Aug 13;12(8):1815. doi: 10.3390/polym12081815 (PMC7464297; doi:10.3390/polym12081815)
Supplement: Supplementary file 1 [file polymers-12-01815-s001.pdf]

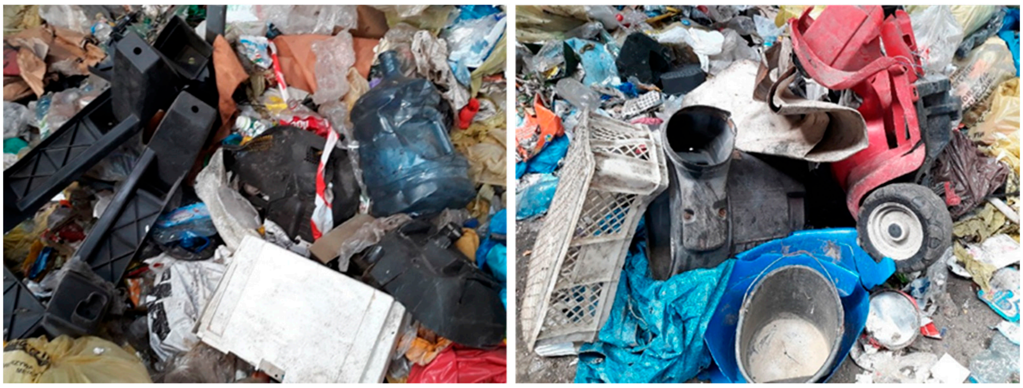

**Figure S1.** Yellow bag waste.

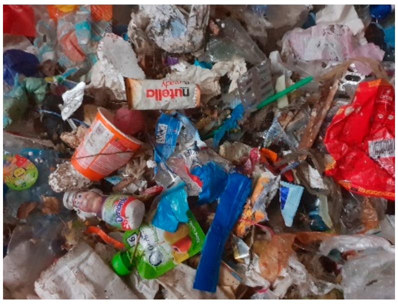
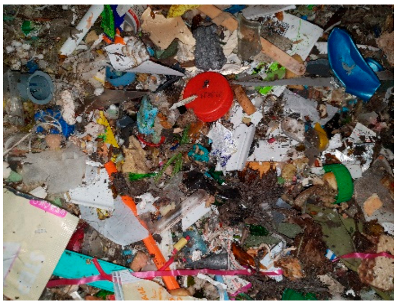
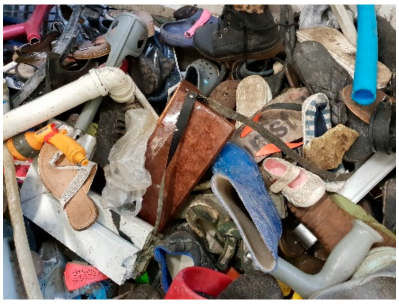

**Fraction <80 mm** **Fraction 0–40 mm** **Waste with chlorine**

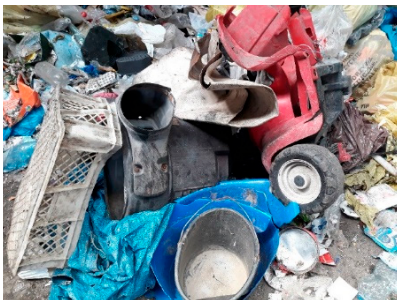
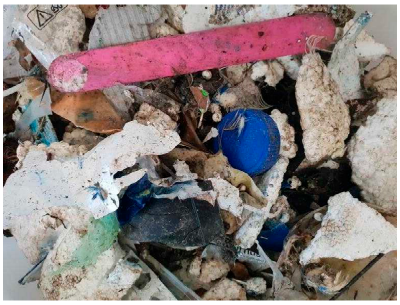
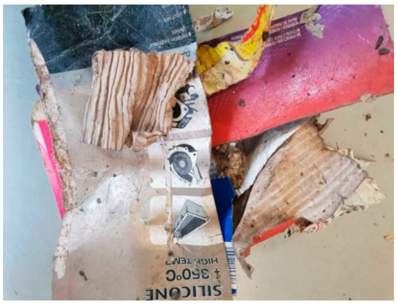

**Balast** **RDF** **Paper mix**

**Figure S2.** Waste fractions separated on the process line.
